# Responsiveness of Health Professionals to Postabortion Care at a Regional Level Hospital in Ghana: A Qualitative Study of Patients' Self-Reports

**DOI:** 10.1155/2018/3861760

**Published:** 2018-05-06

**Authors:** Kenneth Setorwu Adde, Eugene Kofuor Maafo Darteh, Akwasi Kumi-Kyereme, Hubert Amu

**Affiliations:** ^1^Department of Population and Health, College of Humanities and Legal Studies, University of Cape Coast, Cape Coast, Ghana; ^2^Department of Population and Behavioural Sciences, School of Public Health, University of Health and Allied Sciences, Hohoe, Ghana

## Abstract

**Background:**

The responsiveness of health professionals to patients in the provision of abortion services is essential to influencing patients' perceptions and expectations regarding the quality of medical care to be received and their general satisfaction. This, in turn, determines if patients will revisit a particular health facility to access abortion services. In this study, we examine the responsiveness of health professionals in providing postabortion care at a regional level health facility in Ghana.

**Methods:**

Qualitative data collected from 20 female patients who assessed abortion services at a regional level health facility in Ghana were used. The sample was achieved through saturation while a systematic qualitative orientated text analysis was adopted in analysing the data.

**Results:**

Health professionals were responsive to postabortion care at the facility. Most women who sought postabortion care at the facility were referred from other health facilities which could not handle such cases. Other reasons include satisfaction with services received on previous visits to the hospital. We also realized, however, that postabortion services were not covered by the National Health Insurance Scheme.

**Conclusions:**

All hospitals across the country should be equipped with the basic equipment and personnel to conduct and manage abortions. This would reduce not only referrals but also possible maternal deaths. Abortion services should also be added to the services covered by the country's National Health Insurance Scheme.

## 1. Introduction

Globally, about 830 women die from pregnancy and childbirth-related complications every day, and abortion accounts for 8% of these maternal mortality cases [1–3]. Although abortion rates in the developed world have declined significantly since the 1990s, unsafe abortions continue to claim the lives of thousands of women annually in developing countries [4]. In some developing countries, substandard postabortion services have been reported by patients seeking postabortion care (PAC) [5]. They indicate that such substandard services are displeasing and, thus, deter them from seeking subsequent postabortion care in some facilities [6, 7]. PAC refers to emergency treatment services for complications of spontaneous or unsafely induced abortions as well as postabortion family planning counseling and services [7].

In Ghana, abortion is the leading cause of maternal mortality, accounting for about 11% of maternal deaths [8, 9]. Abortion in Ghana is relatively liberal, compared to other Sub-Saharan countries [9, 10]. The abortion law of 1985 (PNDC Law 102), for instance, prohibits the act but states three main conditions under which an abortion can be done [8]. Abortion is allowed when pregnancy results from rape or defilement of a female idiot (who is an imbecile, mad, or under 18 years of age and cannot take decisions for herself) or from incest [8]. It is, also, allowed when the continuance of a pregnancy would involve substantive risk to the life of the pregnant woman or injury to her physical or mental health. Abortion is finally acceptable under Ghana's abortion law when there is a substantial risk that the child, if born, may suffer from or later develop a serious physical abnormality or disease [11, 12].

From a policy perspective, Ghana did not integrate safe abortion into national reproductive health policy until 2003 [8]. In 2006, the Ghana Health Service introduced the new standards and protocols for safe abortion services that include a direction for interpreting Ghana's abortion law. However, to a large extent, the law still tends to be interpreted as prohibiting abortion and, hence, the limited access to abortion services in the public sector [11].

To curb the negative effects of unsafe abortions (which include rupture of the uterus, sepsis, anaemia, and death) in Ghana, the government introduced a comprehensive reproductive health strategy that focuses on maternal morbidity and mortality connected with unsafe abortion [13]. Manual Vacuum Aspiration was, for instance, introduced in the curriculum for midwifery education in the country. However, social, religious, policy, and legal restrictions on abortion continue to serve as barriers to having access to comprehensive abortion care, thereby, forcing women to opt for unsafe abortion [14].

Even though, as a country, Ghana has been increasing efforts to improve access to postabortion care, especially when such abortions are conducted clandestinely, the services have remained underfunded with low visibility and poor quality. As such, they remain inaccessible to most women [15]. Meanwhile, a hospital, be it large or small, can demonstrate successful performance only when it satisfies the factors of quality and satisfaction that a patient expects. The perceptions and expectations of patients regarding the quality of medical care and their general satisfaction are, thus, important in determining if patients will revisit a particular health facility to access abortion services [16]. The responsiveness of the health system and professionals to the provision of services to patients, therefore, becomes very imperative. The World Health Organisation describes responsiveness as “the ability of the health system to meet the population's legitimate expectations regarding their interaction with the health system, apart from expectations for improvements in health or wealth” [17].

Despite the fact that much work has been done by the government to curtail maternal morbidity and mortality as a result of unsafe abortion and postabortion complications, there is a paucity of empirical literature in Ghana with a focus on the responsiveness of health care facilities and professionals to abortion and postabortion care. This study, therefore, examined the responsiveness of health professionals at a regional level health facility to postabortion care from the patients' perspective. An understanding of this issue is an important step towards the implementation of interventions to improve access to PAC in Ghana and other low-resource settings across the globe.

## 2. Conceptual Framework

The study is underpinned by the health system responsiveness model. This model was developed by the WHO with the goal of developing technical tools to assess, monitor, and raise awareness of how patients are cared for and the environment in which they receive such care when seeking health services. The underlining assumption of the framework is that as a social system the health system is expected to meet a core goal as well as a common social goal expected from all social systems [18].

The health system performance measurement is important because it helps identify the gaps in health systems [19]. It, also, provides indicators for examining a health system over a period. Responsiveness under this framework has been defined to encompass the non-health enhancing and the nonfinancial aspects of the health system. According to Smith [19], words that are commonly used in the discussion of responsiveness are satisfaction and quality of care. Patient satisfaction, in this context, includes perceived need, expectations, and experience of care. The quality of care covers a wide dimension; this includes structural quality, process quality, service quality, and interpersonal quality [20, 21]. This model has been adopted because the WHO proposed it as a desirable measure by which health systems can be judged in the context of performance assessment from patients' views on the quality of care provided and satisfaction with such care, which is what the present study sought to investigate [22].

## 3. Materials and Methods

This qualitative study was conducted cross-sectionally. The study was cross-sectional because data were collected from participants only once without the intention of conducting future follow-ups. The data collection was conducted at a regional level health facility in Ghana (Ghana is divided into ten administrative regions for the purposes of governance and development). The selected hospital is a state-owned ultramodern regional referral hospital. Obstetrics and Gynecology is one of the several services provided at the hospital. According to the Ghana Maternal Health Survey of 2007, the highest incidence of induced abortion was recorded in the region [23]. For instance, it was noted that 12.3% of women in the reproductive age group in the region had ever had an abortion [23]. Meanwhile, about 94.6% of abortion seekers in the region reported not being provided with abortion services when they visited public health facilities as they were met with negative attitudes from service providers [24].

With the help of health providers, patients with abortion experiences were purposively selected for the study. The PAC patients who were willing to take part in the study were adequately informed about the study and provided with informed consent forms to sign/thumbprint. The patients who agreed to participate in the study were then allowed to select their own time and place for the interview. The interviews were recorded and transcribed. On the average, an interview spanned about 45 minutes. A sample size of 20 women, achieved through saturation, was used for the study.

A self-developed in-depth interview (IDI) guide was used to collect data from women who received postabortion care at the hospital. The IDI was used to acquire in-depth information from participants within the milieu of personal experiences with postabortion care. The instrument was divided into two sections: A and B. While Section A focused on the background information of participants, Section B dealt with issues related to the responsiveness of health professionals at the hospital. In all, the instrument was made up of 10 items which contained further probes. The validity and trustworthiness of the instrument were achieved by giving the instrument to two experts who perused it and made valuable inputs. Based on their review, two questions which did not appropriately address the objectives of the study were expunged while three more questions were introduced to ensure that all issues that the study sought to address were appropriately covered. This review, also, led to the reconstruction of three questions.

Ethical clearance for the study was obtained from the Ghana Health Service Ethical Review Committee (GHS-ERC: 14/10/15) and the University of Cape Coast Ethical Review Board (UCCIRB/CHLS/2015/07). A permission to conduct the study was, also, obtained from the management of the hospital of concern. The data were analysed manually using a qualitative content analysis technique. A systematic qualitative orientated text analysis was, thus, adopted. The data were coded and themes developed based on both a priori and emergent issues. Finally, quotes from the participants were used to substantiate issues discussed.

## 4. Results

### 4.1. Sociodemographic Characteristics of Participants

Table 1 presents the sociodemographic characteristics of the participants. Eighty percent were in their 20s while 15% were aged 30–39 years. Those who had attained basic education constituted 35% whereas 30% had tertiary education. Ninety-five percent of the participants were Christians while 5% were Muslims. Fifty-five percent of the participants were never married while 45% were married at the time of the study. The main occupations of the participants were dressmaking (25%), civil service (25%), and trading (20%).

### 4.2. Responsiveness to Postabortion Care

The major considerations of participants on the responsiveness of health professionals to postabortion care include prompt attention, the attitude of health professionals, patients' satisfaction, and choice of health professionals. Aside from these, the participants noted the challenges they experienced with PAC, their satisfaction with services, and the attitude of providers and the factors which influenced their decision to seek care at the facility.

#### 4.2.1. Prompt Attention

We realized that the hospital placed priority on giving early care to its patients. There were instances where patients were offered treatment upon arrival and prior to the required initial procedure of registering a patient (documenting necessary information such as name, date of birth, and place of residence) for the service. There were also occasions where health professionals took it upon themselves to process the documents on behalf of the patients while they were treated. For instance, a 25-year-old trader who was treated while a hospital staff was processing her card said the following:

*… Oh, they were quick in attending to me. As soon as I got here, I was sent to the ward for treatment to begin but I can't really tell like how many minutes it took them to attend to me because I was in pain and I was really not thinking about that. I was actually in pain from the house so when I got here the door was not opened by then so I approached a young male nurse and as soon as I told him what was happening to me, he took a wheelchair and wheeled me to the ward and they started treatment on me while he went on ahead to process my card ….* (Trader, 25 years)

Another participant who received prompt attention indicated the following:

*Yes when I got here, they didn't even make me stay at the OPD (outpatient department), I was moved to the gynecological ward and my husband processed the folder at the OPD while I was being treated*. (Civil servant, 23 years)

#### 4.2.2. Attitude of Health Professionals

The attitude of health professionals towards patients is an integral part of postabortion care [6]. This study revealed that the participants were generally happy about the attitude of health professionals at the hospital. The professionals were generally praised for being caring and polite. A 33-year-old farmer who expressed satisfaction with the attitude of the professionals said the following:

*Oh, they are good, caring and are free with us. For some other hospitals, the nurses are not friendly at all and will fight and argue with you at the least thing. But at this hospital, they are very kind and free. Ever since I came all those who came to speak with me talk with some kind of respect for me and if they say something or do something and realized you are not happy, they are quick to apologize.* (Farmer, 33 years)

Another participant indicated the following:

*Their attitude, in general, is fine. They know how to talk to us and are not abusive in their choice of words and they take very good care of us like coming to check up on us regularly to see if everything is going on well.* (Seamstress, 23 years)

About one-fourth of the participants, however, felt the health professionals exhibited negative attitudes. The negative attitudes they complained about include insulting and provocative behaviour of the health professionals. A 28-year-old hairdresser said the following:

*I will not say everyone is having a good character. When I was here the other time, they really worried me. Is it insult they did not insult me? But I was in pain so I couldn't talk but those who treated me yesterday were very nice.* (Hairdresser, 28 years)

Corroborating the negative attitude of the health professionals, another participant noted the following:

*Is this a good hospital? This is just a place for insulting. Hmmm the people are wicked and they don't take care of me. I have not been to a hospital that they have treated me like that before. The people are wicked, they are very wicked papa. Even if you are dying they won't mind you.* (Student, 28 years)

#### 4.2.3. Challenges

Most of the women reported to the hospital with little or no money with the intention of using their health insurance cards. Ghana operates a National Health Insurance Scheme (NHIS) which provides services to residents free of any charges at the point of service delivery after payment of annual premiums [25]. Exemptions are, however, made with regard to such payments for children under 18 years, social security and national insurance trust contributors and pensioners, pregnant women, and the indigent [26]. However, the women only got to know at the facility that the insurance did not cover their treatments. A participant who was disappointed said the following:

*The only thing that is bothering me is that I had only GH*₵*30 when I was coming knowing very well the health insurance will also cover some costs but they are telling me I have to pay GH*₵*130 and that the insurance card does not cover everything. She added that if not for the card, I would have paid about GH*₵*400.* (Hairdresser, 28 years)

Another disappointed participant noted the following:

*Well, the government told us that health insurance will help us when we come to the hospital but when you come, then they expect you to pay for the medicine again and when you are done and about to leave they give you bill again and I don't understand why it should be like that. For example, when they wanted to clean my stomach (evacuation of the womb) for me, I was asked to pay for it as well as other things like water and medicines, which I think should be covered by the NHIS.* (Seamstress, 23 years)

### 4.3. Patients Satisfaction with Postabortion Care

More than three-quarters of the women expressed satisfaction with the postabortion care services at the facility. Overall, the women expressed satisfaction mainly because of the attitude of the health professionals towards them and how they were catered for even when they had no money to pay for the service. The participants said the following about the attitudes of the health professionals towards them:

*The reason I am very satisfied is that I have been to different hospitals and I have come here too and I have seen the difference. Surprisingly, this happens to be the hospital that I was rather afraid of coming to, but I am really glad I did come here. The health workers here are very nice and caring towards us.* (Trader, 29 years)

Another participant said

*… they are quick to attend to patients. Unlike other places that when you go and you are in pain, they will just stand and be watching you and how to even attend to patients or give them treatment becomes a problem. But for the three days I spent at the regional hospital, I was amazed at the way everyone who comes is dedicated to their work. Everyone was serious about their work and did it to satisfaction at the same time smiling at us*. (Farmer, 33 years)

Others also expressed satisfaction with the treatment they received mainly because after the treatment they did not suffer any complications again. For instance, a satisfied participant indicated the following:

*Yes because the day they cleaned my stomach, the following day and I was okay and I did not feel any pain again but some other sister came and she really suffered after the process. I think she even got a swollen thigh.* (Student, 19 years)

#### 4.3.1. Factors Influencing Patients' Choice of Facility

The major factors which influenced participants' choice of the facility were referrals from other facilities and past experiences. Regarding referrals, the women noted that, to a large extent, they did not decide on the facility to receive their postabortion care by themselves. Most of them were referred from other health facilities where they first sought PAC services. For instance, a participant who was referred from another health facility said the following:

*… I was then sent to a hospital in Togo. When we got there, they pressed my stomach for long before it came out and I was in severe pain. But they said they were not having the machine that they will use to clean my stomach so they referred me to the VRH.* (Hairdresser, 28 years)

To corroborate this statement, another participant indicated the following:

*I was sent to the hospital in Sokode. They managed to stabilise my condition there and then transferred me to the regional hospital. It was at the regional hospital that they carried out some tests on me and detected that I was pregnant but the pregnancy got spoilt (had spontaneous abortion) so went ahead to clean my stomach for me (evacuation of the uterus).* (Farmer, 33 years)

Some of the women, however, indicated that their choice of the facility was influenced by their past experiences at the hospital. Such experiences include the treatment and the care they received while at the hospital. A participant with a past experience from the Volta Regional Hospital indicated the following:

*Well! this is the hospital that I had my first child and I liked how I was treated here so I don't see any reason to go anywhere else. Even my first child, I decided to come here because they treat people well. Even if you come alone and have no one to aid you, they themselves will take up that responsibility and take care of you for you to be comfortable. For example, when you come to this hospital and you need to purchase some things, but you don't have money, they will give it to you and you pay for it later.* (Hairdresser, 23 years)

Another participant said the following:

*Because I don't have anyone who will take care of me at the hospital so I decided to come to the regional hospital. Unlike other hospitals that you need a relative or someone to be coming to wash for you, take care of you among other things, this place, the nurses do all that for you and make sure you are always comfortable.* (Farmer, 37 years)

## 5. Discussion

An essential factor to consider when assessing the quality of care of a health facility is the viewpoint of the patient. Since patients' needs often vary, their personal satisfaction rests on individual views and expectations [27]. Our findings show that waiting time for treatment at the hospital was relatively short: the women were satisfied with the prompt attention they received at the facility. This according to the conceptual framework is part of patient orientation which offers prompt attention to the patient [28]. This is, however, contradictory to findings made by Tesfaye and Oljira [29] who observed that the waiting time from arrival to treatment is usually very long. Nonetheless, other studies revealed that there is a bias in the delivery of early treatment to patients, with adolescents often not given early attention [30].

In line with postulations of the health system responsiveness, patients are expected to be treated with respect in terms of dignity, autonomy, and confidentiality [28]. We realized in our study that patients were generally satisfied with postabortion care at the hospital mainly because of positive attitudes exhibited towards them by the health professionals. These findings are consistent with that of Melkamu et al. [5] who reported that health professionals are usually caring and polite to patients who sought PAC services. However, some expressed dissatisfaction with PAC services at the hospital due to the provocative nature of some health professionals. This, also, affirms the findings of Arambepola et al. [31] in Sri Lanka, arguing that postabortion patients were displeased with services received from health facilities owing to the verbal harassment meted out to them by some health care providers due to their abortion condition.

The findings, however, show that treating patients with care and respect at a health facility gives them the needed satisfaction with health care services. The health professionals at the hospital were, therefore, applauded by the participants for their attitude towards them. Melkamu et al. [6] opined that most women perceive postabortion services in health facilities to be satisfactory and are, therefore, motivated to access postabortion services in hospitals. The specific factors that prompted this positive perception include the value of care, good conduct of health professionals, and the proximity of the health facilities [5, 6]. This corroborates the current findings as well as the health system responsiveness model which requires health systems to provide structural quality, service quality, and interpersonal quality to attain patient satisfaction [21].

The inability of some hospitals to perform postabortion care services resulted in the referral of some participants to the hospital. Others, however, noted that they chose the facility for the services mainly based on their past experiences at the hospital which they considered satisfactory. This affirms the health systems' responsiveness model's proposal that the ability to exercise preferences could be as a result of treatment by persons of a particular sex, age group, and emotional bonding to a particular facility [18]. This, also, agrees with the findings of Wariki [32] who argued that patients seek postabortion care at particular hospitals as a result of their satisfaction with the services at the facility and, hence, their enthusiasm to return for a follow-up and recommend the services to others.

Even though participants had subscribed to the NHIS, they realized at the facility that the scheme did not cover their treatments. This points to the challenges and concerns that have been raised by subscribers concerning the inadequacies of the health insurance over the years. The exclusion of abortion and postabortion services from the scheme may result in needless deaths [33]. Meanwhile, abortion services are considered basic services due to the high possibility of maternal deaths if they are not handled early and appropriately [34].

Despite the important findings made from this study, there are possible limitations inherent in the study that are worth mentioning. For instance, the fact that the study was conducted in only one facility and used a relatively small sample size greatly limits its generalizability to the general Ghanaian society. This limitation, however, does not limit the reliability and trustworthiness of the study in any way as the necessary steps needed to make the instrument valid and trustworthy were adhered to. Also, the study was conducted cross-sectionally. Trends in the responsiveness of the facility may, however, change in future as such trends may differ from current findings made in our study.

## 6. Conclusion

We established that health professionals were responsive to postabortion care at the facility. Patients were offered early treatment at the hospital. PAC patients were also found to be generally satisfied with postabortion care services at the facility. Women sought postabortion care at the facility generally as a result of referrals from other health facilities that could not handle such cases and also based on satisfaction with services received on previous visits to the hospital.

The referral of postabortion cases by some facilities could result in complications with serious maternal health implications for the patients. Pragmatic steps should be taken by the Ministry of Health and Ghana Health Service to equip all health facilities across the country with the needed facilities and personnel with the requisite skills to provide complete postabortion care. Also, efforts should be made by the NHIA to add postabortion care to the range of services covered by the NHIS.

## Figures and Tables

**Table 1:** Socio-demographic characteristics of participants.

Variable	Frequency	Percentage (%)
*Age (In completed years)*		
<20	1	5.0
20–29	16	80.0
30–39	3	15.0
*Level of education*		
No education	2	10.0
Primary	3	15.0
JHS	7	35.0
SHS	2	10.0
Tertiary	6	30.0
*Religion*		
Christian	19	95.0
Muslim	1	5.0
*Marital status*		
Never married	11	55.0
Married	9	45.0
*Occupation*		
Dressmaking	5	25.0
Trading	4	20.0
Farming	2	10.0
Civil service	5	25.0
Schooling	2	10.0
Hairdressing	2	10.0

*Total* ^*∗*^	*20*	*100.0*

*Source*. Fieldwork, 2016 ^**∗**^Total represents the 20 participants which were recruited for the study.
